# Association of Enlarged Perivascular Spaces and Total Small Vessel Disease Burden With Kidney Function

**DOI:** 10.1212/WNL.0000000000218406

**Published:** 2026-08-04

**Authors:** Philip S. Nash, Gareth Ambler, Jonathan G. Best, Duncan Wilson, Houwei Du, Rustam Al-Shahi Salman, Hans Rolf Jäger, Gregory Y.H. Lip, Martina B. Goeldlin, Morin Beyeler, Philipp Bücke, Marwan El-Koussy, Heinrich P. Mattle, Leonidas Panos, Dianne H.K. van Dam-Nolen, Florian Dubost, Jeroen Hendrikse, M. Eline Kooi, Werner H. Mess, Paul J. Nederkoorn, Nicolas Christ, Maximilian Bellut, Sarah Gunkel, Christopher Charles Karayiannis, John Ly, Shaloo Singhal, Lee-Anne Slater, Young Dae Kim, Keon-Joo Lee, Jae-Sung Lim, Hideo Hara, Masashi Nishihara, Jun Tanaka, Masaaki Yoshikawa, Derya Selcuk Demirelli, Zeynep Tanriverdi, Ender Uysal, Shelagh B. Coutts, Francesca M. Chappell, Stephen DJ Makin, Henry Ka Fung Mak, Kay Cheong Teo, Debbie Y.K. Wong, Lisa Hert, Marta Kubacka, Philippe A. Lyrer, Alexandros A. Polymeris, Benjamin Wagner, Annaelle Zietz, Jill Abrigo, Cyrus Cheng, Winnie Cw Chu, Thomas W. Leung, David Julian Seiffge, Urs Fischer, Simon Jung, Daniel Bos, Felix Fluri, Thanh G. Phan, Velandai K. Srikanth, Ji Hoe Heo, Hee-Joon Bae, Yusuke Yakushiji, Dilek Necioglu Orken, Eric E. Smith, Joanna M. Wardlaw, Gary Kui Kai Lau, Stefan T. Engelter, Nils Peters, Yannie O.Y. Soo, Tae-Jin Song, Robert J. Simister, David C. Wheeler, David J. Werring

**Affiliations:** 1 UCL Stroke Research Centre, Department of Translational Neuroscience and Stroke, University College London Queen Square Institute of Neurology, United Kingdom;; 2 Comprehensive Stroke Service, National Hospital for Neurology and Neurosurgery, University College London Hospitals NHS Trust, United Kingdom;; 3 Department of Statistical Science, University College London, United Kingdom;; 4 Department of Neurology, Fujian Medical University Union Hospital, Fuzhou, China;; 5 Institute for Neuroscience and Cardiovascular Research at the University of Edinburgh, United Kingdom;; 6 Lysholm Department of Neuroradiology and the Neuroradiological Academic Unit, Department of Translational Neuroscience and Stroke, UCL Queen Square Institute of Neurology, London, United Kingdom;; 7 Liverpool Centre for Cardiovascular Science at the University of Liverpool, Liverpool John Moores University and Liverpool Heart and Chest Hospital, United Kingdom;; 8 Department of Clinical Medicine, Aalborg University, Denmark;; 9 Department of Cardiology, Lipidology and Internal Medicine with Intensive Coronary Care Unit, Medical University of Bialystok, Poland;; 10 Department of Neurology, University Hospital Inselspital Bern, University of Bern, Switzerland;; 11 Department of Diagnostic and Interventional Neuroradiology, University Hospital Inselspital Bern, University of Bern, Switzerland;; 12 Department of Radiology and Nuclear Medicine, Erasmus MC, Rotterdam, the Netherlands;; 13 Biomedical Imaging Group Rotterdam, Department of Radiology and Nuclear Medicine, Erasmus University Medical Centre, Rotterdam, the Netherlands;; 14 Department of Radiology, University Medical Centre Utrecht, Utrecht University, the Netherlands;; 15 Department of Radiology and Nuclear Medicine, CARIM Cardiovascular Research Institute Maastricht, Maastricht University Medical Center (MUMC+), the Netherlands;; 16 Department of Clinical Neurophysiology, Maastricht University Medical Centre, the Netherlands;; 17 Department of Neurology, Amsterdam University Medical Centres, Location AMC, the Netherlands;; 18 Department of Neurology, University Hospital of Würzburg, Germany;; 19 Peninsula Clinical School, Peninsula Health, Monash University, Melbourne, Australia;; 20 Stroke and Ageing Research Group, School of Clinical Sciences at Monash Health, Monash University, Melbourne, Australia;; 21 Department of Interventional Radiology, Monash Health, Clayton, Victoria, Australia;; 22 Department of Neurology, Yonsei University College of Medicine, Seoul, South Korea;; 23 Department of Neurology, Seoul National University Bundang Hospital, Seoul National University College of Medicine, Seongnam, Republic of Korea;; 24 Department of Neurology, Asan Medical Center, University of Ulsan College of Medicine, Seoul, Republic of Korea;; 25 Division of Neurology, Department of Internal Medicine, Saga University Faculty of Medicine, Nabeshima, Japan;; 26 Department of Radiology, Saga University Faculty of Medicine, Nabeshima, Japan;; 27 Department of Neurology, Sisli Hamidiye Etfal Teaching and Research Hospital, University of Health Sciences, Istanbul, Turkey;; 28 İzmir Katip Çelebi University Atatürk Education and Research Hospital Department of Neurology, Izmir, Turkey;; 29 Saglık Bilimleri University Sisli Etfal Education and Research Hospital Department of Radiology, Istanbul, Turkey;; 30 Calgary Stroke Program, Department of Clinical Neurosciences, Radiology and Community Health Sciences, Hotchkiss Brain Institute, University of Calgary, Canada;; 31 Centre for Clinical Brain Sciences, Edinburgh Imaging, University of Edinburgh, United Kingdom;; 32 UK Dementia Institute at the University of Edinburgh, United Kingdom;; 33 Institute of Applied Health Sciences, University of Aberdeen, United Kingdom;; 34 Department of Diagnostic Radiology, The University of Hong Kong, Hong Kong;; 35 Division of Neurology, Department of Medicine, The University of Hong Kong, Hong Kong;; 36 Department of Neurology and Stroke Centre, University Hospital Basel and University of Basel, Switzerland;; 37 Stroke Center Klinik Hirslanden, Zürich, Switzerland;; 38 Department of Imaging and Interventional Radiology, Prince of Wales Hospital, The Chinese University of Hong Kong, Hong Kong;; 39 Department of Medicine and Therapeutics, Prince of Wales Hospital, The Chinese University of Hong Kong, Hong Kong;; 40 Department of Neurology, Kansai Medical University, Hirakata, Osaka, Japan;; 41 Department of Neurology, İstanbul Nişantaşı University, Turkey;; 42 Neurology and Neurorehabilitation, University Department of Geriatric Medicine FELIX PLATTER, University of Basel, Switzerland;; 43 Department of Neurology, Seoul Hospital, Ewha Womans University College of Medicine, Seoul, South Korea; and; 44 Department of Renal Medicine, University College London, United Kingdom.

## Abstract

**Background and Objectives:**

Enlarged perivascular spaces (EPVSs) in the basal ganglia (BG-EPVS) are an important marker of cerebral small vessel disease (cSVD), and EPVS in the centrum semiovale (CSO-EPVS) are part of the diagnostic criteria for cerebral amyloid angiopathy. We aimed to investigate associations of EPVS with reduced estimated glomerular filtration rate (eGFR) and glomerular hyperfiltration (higher than normal eGFR), which have scarcely been studied previously.

**Methods:**

In this cross-sectional study, we used pooled individual patient data from the Microbleeds International Collaborative Network which includes patients with ischemic stroke or transient ischemic attack. We investigated associations of impaired kidney function, defined as an eGFR of 30–60 or <30 mL/minute/1.73 m^2^, and glomerular hyperfiltration, defined as eGFR above the age-adjusted and sex-adjusted 95th centile, with BG-EPVS and CSO-EPVS severity. EPVS were rated according to a validated 5-point ordinal scale, and combined cSVD burden was rated using a validated 5-point ordinal scale with 1 point assigned for the presence of each of the following: severe white matter hyperintensities, ≥1 cerebral microbleed, ≥1 lacune, and BG-EPVS ≥11. Normal glomerular filtration was defined as eGFR ≥60 without hyperfiltration. We used multivariable ordinal logistic regression models to estimate risk of increased EPVS and cSVD burden severity adjusted for age, sex, and comorbidities.

**Results:**

Seven thousand two hundred fifty-four patients (mean age 71 ± 13 years, 43% female) were included in the analysis, 357 with glomerular hyperfiltration, 1,692 with eGFR 30–60, and 256 with eGFR <30. Compared with normal glomerular filtration, hyperfiltration was independently associated with BG-EPVS (adjusted odds ratio [aOR] 1.38, 95% CI 1.11–1.70, *p* < 0.001) and CSO-EPVS (aOR 1.34, 95% CI 1.08–1.64, *p* = 0.011). Associations of eGFR 30–60 and eGFR <30 with EPVS were not statistically significant. Compared with normal glomerular filtration, eGFR <30 (aOR 1.27, 95% CI 1.03–1.57) was independently associated with increased cSVD burden, but eGFR 30–60 (aOR 1.06, 95% CI 0.95–1.20) and hyperfiltration (aOR 1.15, 95% CI 0.98–1.34) were not.

**Discussion:**

Glomerular hyperfiltration was independently associated with EPVS severity, in both the basal ganglia and centrum semiovale. eGFR <30 was independently associated with total cSVD burden. A key limitation was a lack of repeated eGFR measurements.

## Introduction

Perivascular spaces are narrow fluid-filled spaces following the typical course of small perforating cerebral arteries,^1^ with similar signal intensity to CSF on all magnetic resonance (MR) sequences. They are thought to be an important part of the cerebral glymphatic system and responsible for returning breakdown products of cellular metabolism to the circulation as part of cerebral homeostasis.^2^ Enlarged perivascular spaces (EPVS) are a neuroimaging marker of impaired glymphatics and are associated with pathologic blood-brain-barrier (BBB) permeability.^3^ They are a recognized marker of cerebral small vessel disease (cSVD), as per STRIVE-2 consensus definitions.^1^ EPVS in the basal ganglia (BG-EPVS) are associated with systemic vascular risk factors such as age and hypertension^4^ and have been shown to be associated with the risk of recurrent stroke in a large international collaborative cohort study.^5^ EPVS in the centrum semiovale (CSO-EPVS) are a histologically proven feature of cerebral amyloid angiopathy^6^ (CAA) and are part of the current diagnostic criteria.^7^ However, they have not been consistently demonstrated to be associated with stroke risk.^5,8^

Chronic kidney disease (CKD) is a recognized risk factor for cerebrovascular disease, including ischemic stroke,^9,10^ intracerebral hemorrhage^11,12^ (ICH), and cSVD.^13^ Associations of cSVD and CKD have been demonstrated in community populations and stroke populations. However, most of these studies concern cerebral microbleeds (CMB) and white matter hyperintensities (WMH), with just a handful of studies investigating associations of EPVS with CKD in stroke populations.^14-16^ These studies had discordant results, with Xiao et al.^16^ reporting a strong association of estimated glomerular filtration rate (eGFR) < 60 mL/minute/1.73 m^2^ with the presence of severe EPVS in both the basal ganglia and centrum semiovale, and the other studies^14,15^ reporting no association. Therefore, this is an area of research which has not yet been completely characterized. Similarly, associations of CKD and the overall burden of cSVD have only been investigated in a small number of studies.^14,16-18^ Since BG-EPVS have mainly been associated with cSVD markers of arteriolosclerosis (deep ICH,^19^ lacunar stroke,^20^ deep CMB,^5^ and WMH^20^), and CKD has also primarily been associated with arteriolosclerosis^12^; CKD might plausibly be associated with BG-EPVS. Conversely, since rates of CKD in those with CAA-related ICH have been demonstrated to be low (14% in a recent ICH registry study^21^), one might not expect an association of CKD with CSO-EPVS.

cSVD and renal microvascular disease can have severe clinical consequences and could be part of a shared pathophysiologic process.^22^ The renal filtration barrier and blood-brain barrier (BBB) share some similar structural and functional properties, with cellular architecture organized in a broadly similar orientation: specialized endothelial cells enmeshed with pericytes and astrocyte foot processes in the BBB^23^ and mesangial cells and podocyte foot processes in the glomerulus.^24^ Both structures provide a selectively permeable barrier to proteins, ions, and solutes. If dysfunction of both of these barriers developed in parallel, this would support the hypothesis that a shared multisystem endotheliopathy is an important mechanism of injury.

Glomerular hyperfiltration, or higher than normal eGFR, is an earlier marker of glomerular endothelial injury than overt CKD,^25^ and it is emerging as a novel vascular risk factor.^26-28^ It occurs as a compensatory mechanism for nephron loss, most commonly in the context of diabetic nephropathy.^29^ Overstimulation of the renin-angiotensin-aldosterone (RAA) axis leads to increased glomerular perfusion and increased intraglomerular pressure. In the long term, this effect is deleterious and leads to further nephron loss and the development of reduced eGFR and CKD. To the best of our knowledge, there is no published scientific evidence investigating glomerular hyperfiltration with cSVD.

Therefore, we aimed to investigate associations of impaired kidney function and glomerular hyperfiltration with the presence and severity of EPVS and overall cSVD burden, in a large international collaboration of pooled individual patient data. We hypothesized that those with impaired kidney function and those with renal hyperfiltration might have more severe EPVS than those with normal filtration.

## Methods

This is a cross-sectional analysis of the baseline characteristics and neuroimaging data of contributing centers from the Microbleeds International Collaborative Network (MICON). This study is reported according to Strengthening the Reporting of Observational Studies in Epidemiology (STROBE) guidelines.

### Patient Selection

Studies were eligible for the inception MICON individual patient data meta-analysis if they included at least 50 adult (>18 years) patients with ischemic stroke or TIA, had MRI data including blood-sensitive sequences, and collected recurrent stroke outcomes prospectively for at least 3 months and up to 5 years. Participants from the original collaboration were eligible for inclusion in this study if their study centers elected to contribute data on perivascular spaces and kidney function, taken during the index hospitalization. Participants were excluded if they were missing data on either of these variables or if other demographic information needed to determine eGFR were missing.

### Classification of Glomerular Function and eGFR Estimation

We classified the study population according to eGFR. Glomerular hyperfiltration was defined as an eGFR above the age-adjusted and sex-adjusted 95th centile.^26,30^ Mild to moderate impaired kidney function was defined as eGFR 30–60 mL/minute/1.73 m^2^, and severe impaired kidney function was defined as eGFR <30, in line with recommendations from Kidney Disease: Improving Global Outcomes^31^ (KDIGO). To estimate eGFR, we chose to use the 2009 Chronic Kidney Disease Epidemiology Collaboration (CKDEPI) equation^32^ omitting the ethnicity coefficient, as recommended by the European Renal Association^33^ and the European Federation of Clinical Chemistry and Laboratory Medicine.^34^ Creatinine values were considered implausible if they were <20 or >2000 mL/minute.

### Outcomes

The prespecified primary study outcome was EPVS separately in the basal ganglia and centrum semiovale, according to eGFR category. Secondary outcomes included the combined cSVD burden score and its individual components not included in the primary outcome (severe WMH [Fazekas ≥2]; presence of ≥1 lacune; presence of ≥1 CMB). The study protocol was written a priori and is available in the eSAP (statistical analysis plan; note that subsequent to protocol development we changed the definition of glomerular hyperfiltration based on further literature review).

### Neuroimaging Rating Procedures

EPVS were rated according to a validated 5-point ordinal scale,^35^ using the highest count from a single hemisphere and a single MR slice, classifying EPVS burden as 0, 1–10, 11–20, 21–40, and >40. All ratings were performed by trained raters at participating centers, and all raters were trained using a standardized handbook distributed by the coordinating center.^36^ Because of the small numbers of participants with zero and >40 EPVS, we combined categories to classify participants as 0–10, 11–20, and ≥21. WMH were rated using the Fazekas Scale^37^ (1 site used the Age-Related White Matter Changes scale,^38^ and these ratings were harmonized with Fazekas), and CMB were rated using the Microbleed Anatomical Rating Scale^39^ (MARS). The combined cSVD burden score was determined following a commonly-used definition,^40^ with 1 point assigned for each of the following features: severe WMH (Fazekas ≥2), presence of ≥1 lacune, presence of ≥1 CMB, and severe EPVS in the basal ganglia (≥11).

### Statistical Analysis

We described participant characteristics of the eGFR groups using either mean (SD) or median (interquartile range, IQR) for numerical variables, depending on their distributions. We described categorical variables using number (%).

For the primary outcome, we investigated independent associations of EPVS and eGFR group by fitting univariable and multivariable ordinal logistic regression models with EPVS score as the outcome variable, and eGFR group and potential confounders as the predictor variables. Variables which are plausibly associated with renal impairment or cSVD were included in the multivariable models, including age, sex, East Asian study center, hypertension, diabetes, ischemic heart disease, smoking, and hypercholesterolemia. East Asian study center was included in the models as a proxy for ethnicity, as we did not have detailed data on ethnicity. We used the same approach to investigate associations of cSVD burden with eGFR group, using cSVD burden as the dependent variable and the same independent variables as above. We adjusted for clustering within study centers using cluster-robust variance (sandwich) estimators. The eGFR <30 (n = 256) and hyperfiltration (n = 357) groups were relatively small compared with the other groups, although in absolute terms, they were reasonably large. Furthermore, all outcome-eGFR combinations had a reasonable number of patients (e.g., 42 patients were in the BG-PVS > 20 × eGFR < 30 group, the smallest subgroup), so consequently there were no obvious problems with model fitting.

Since the assessment of the proportional odds assumption indicated that it was violated when including age and East Asian study center in the models, we fitted additional multinomial and logistic regression models as sensitivity analyses. Since we only had one-off measurements of eGFR, we conducted an additional sensitivity analysis excluding those with eGFR >120 mL/minute/1.73 m^2^ to assess the impact of potential measurement bias.

We conducted subgroup analyses according to age group (18–60, 61–70, 71–80, >80), sex, index stroke etiology (according to Trial of Org 10172 in Acute Stroke Treatment [TOAST] classification^41^), and characteristics previously associated with CKD and cSVD (hypertension, diabetes, and smoking). To assess the potential impact of differences between study site in more detail, we fitted a random effects model to the complete case dataset as an additional sensitivity analysis. This indicated substantial differences between study sites, so we investigated this further by conducting an additional subgroup analysis according to study site. For each subgroup analysis, we fitted interaction terms between eGFR category and the subgroup of interest.

We assessed potential selection bias by comparing the characteristics of included and excluded participants. We assessed collinearity between all variables included in our multivariable models using variance inflation factors (VIFs). We accounted for missing values using multiple imputation with chained equations (50 imputations). All statistical tests were 2-sided, and the results considered statistically significant at the 5% level. Statistical analyses were performed using Stata (version 18.1, Statacorps).

### Standard Protocol Approvals, Registrations, and Patient Consents

The research received authorization from the UK Health Research Authority (reference number 8/HRA/0188). Each of the included cohort studies had previously secured the necessary ethical and regulatory approvals as per their respective local guidelines. The data shared for this pooled analysis of individual patient data were completely anonymized, ensuring that the identification of individual participants was not possible. Consequently, the need to obtain specific consent from each participant for this analysis was waived by the research ethics committee.

### Data Availability

Data sharing requests will be considered from appropriately qualified researchers. A data sharing agreement must be put in place before any data are shared. Written proposals will be assessed by the members of the MICON steering committee.

## Results

**Figure 1:**
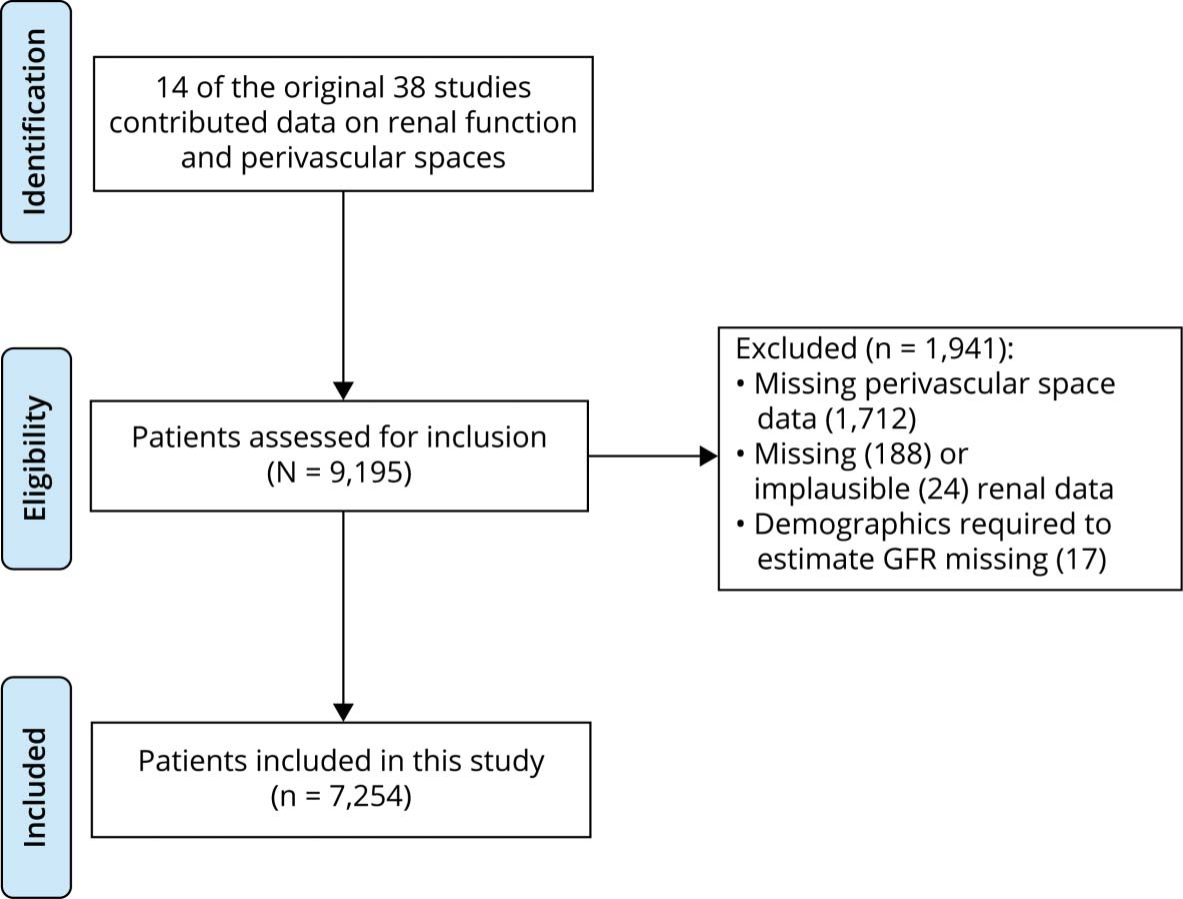
Study Flowchart of Patient Selection GFR = glomerular filtration rate.

Fourteen of the study centers contributing to the original MICON individual-patient data meta-analysis were able to provide data on kidney function and EPVS, contributing 9,195 patients for assessment. Of these 7,254 patients were included in the analysis (mean age 71 ± 13 years, 43% female). Reasons for exclusion included a lack of EPVS data, missing (n = 188) or implausible (n = 24) renal data, and missing demographics required for the CKDEPI estimation equation (n = 17, Figure 1). Compared with included participants, excluded participants were slightly younger (mean age 69 vs 71), had more large artery atherosclerosis (27% vs 20%) and fewer cardioembolic strokes (35% vs 46% as their index stroke etiology, and more came from East Asian study centers (86% vs 54%), but otherwise, there were no substantial differences (eTable 1).

Compared with the normofiltration group, the hyperfiltration group was slightly older (mean age 71 vs 68) and the eGFR 30–60 and eGFR <30 groups were considerably older (78 and 76 years, respectively). Compared with the normofiltration group, there was a similar rate of hypertension in the hyperfiltration group (67% vs 66%) and higher rates in the hypofiltration groups (82% for the eGFR 30–60 group and 87% for the eGFR <30 group). Compared with normofiltration, there were higher rates of diabetes in all other filtration categories, particularly eGFR <30 (48%). Full baseline characteristics of the included patients are presented in Table 1.

**Table 1:** Baseline Clinical Characteristics and Enlarged Perivascular Spaces According to Filtration Group

	All patients (n = 7,254)	Hyperfiltration (n = 357)	Normofiltration (n = 4,949)	eGFR 30–60 (n = 1,692)	eGFR <30 (n = 256)
Age; y; mean (SD)	70.8 (12.5)	70.9 (13.4)	68.2 (12.5)	77.6 (9.4)	76.4 (10.7)
Female sex, (%)	3,113 (42.9)	152 (42.6)	1942 (39.2)	888 (52.5)	131 (51.2)
World region, (%)					
Europe/America	3,368 (46.4)	165 (46.2)	2,180 (44.0)	935 (55.3)	88 (34.4)
East Asia	3,886 (53.6)	192 (53.8)	2,769 (56.0)	757 (44.7)	168 (65.6)
Hypertension	5,093 (70.5)	234 (65.7)	3,251 (65.9)	1,386 (82.3)	222 (87.1)
Diabetes	1795 (25.7)	94 (27.1)	1,086 (23.0)	492 (29.8)	123 (48.2)
Ischemic heart disease	1,059 (14.6)	37 (10.4)	604 (12.2)	348 (20.6)	70 (27.3)
Hyperlipidemia	2,716 (38.9)	109 (31.5)	1774 (37.5)	726 (44.0)	107 (42.0)
Current smoker	1,120 (17.2)	65 (20.8)	846 (19.1)	185 (12.0)	24 (9.8)
eGFR; mean (SD)	74.1 (22.9)	103.2 (14.1)	83.6 (13.9)	48.3 (8.1)	20.1 (8.2)
Index event, (%)					
TIA	725 (10.0)	34 (9.5)	499 (10.1)	175 (10.3)	17 (6.6)
Ischemic stroke	6,529 (90.0)	323 (90.5)	4,450 (89.9)	1,517 (89.7)	239 (93.4)
TOAST classification, (%)					
LAA	1,224 (20.2)	74 (26.6)	933 (22.5)	169 (12.0)	48 (21.2)
Cardioembolic	2,770 (45.8)	110 (39.6)	1731 (41.8)	827 (58.9)	102 (45.1)
Small vessel disease	864 (14.3)	35 (12.6)	664 (16.0)	133 (9.5)	32 (14.2)
Other known cause	255 (4.2)	8 (2.9)	197 (4.8)	44 (3.1)	6 (2.7)
Unknown cause	937 (15.5)	51 (18.3)	617 (14.9)	231 (16.5)	38 (16.8)
Basal ganglia EPVS, (%)					
None	583 (8.0)	31 (8.7)	387 (7.8)	142 (8.4)	23 (9.0)
1–10	3,880 (53.5)	165 (46.2)	2,813 (56.8)	794 (46.9)	108 (42.2)
11–20	1872 (25.8)	110 (30.8)	1,201 (24.3)	478 (28.3)	83 (32.4)
21–40	690 (9.5)	44 (12.3)	418 (8.4)	194 (11.5)	34 (13.3)
>40	229 (3.2)	7 (2.0)	130 (2.6)	84 (5.0)	8 (3.1)
Basal ganglia EPVS, median (IQR)	1 (1–2)	1 (1–2)	1 (1–2)	1 (1–2)	1 (1–2)
Centrum semiovale EPVS, (%)					
None	778 (10.7)	41 (11.5)	475 (9.6)	227 (13.4)	35 (13.7)
1–10	2,222 (30.6)	84 (23.5)	1,497 (30.2)	570 (33.7)	71 (27.7)
11–20	2,383 (32.9)	114 (31.9)	1,690 (34.1)	502 (29.7)	77 (30.1)
21–40	1,637 (22.6)	111 (31.1)	1,128 (22.8)	329 (19.4)	69 (27.0)
>40	234 (3.2)	7 (2.0)	159 (3.2)	64 (3.8)	4 (1.6)
CSO EPVS, median (IQR)	2 (1–3)	2 (1–3)	2 (1–3)	2 (1–2)	2 (1–3)
Total EPVS, median (IQR)	3 (2–4)	3 (2–5)	3 (2–4)	3 (2–4)	3 (2–5)

Abbreviations: CSO = centrum semiovale; eGFR = estimated glomerular filtration rate; EPVS = enlarged perivascular spaces; IQR = interquartile range; LAA = large artery atherosclerosis; TIA = transient ischemic attack; TOAST = Trial of Org 10172 in Acute Stroke Treatment.

### EPVS According to Glomerular Filtration

**Figure 2:**
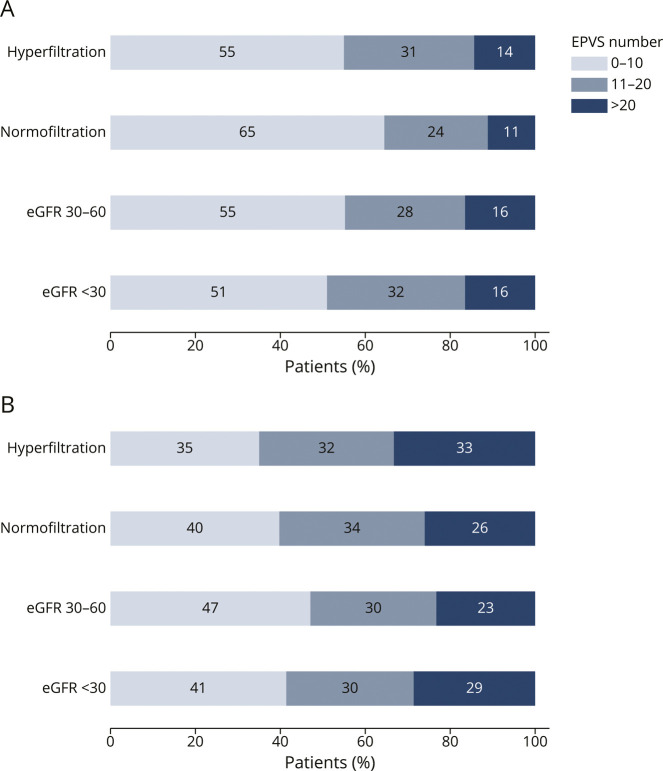
EPVS Severity According to Glomerular Function in (A) the Basal Ganglia and (B) the Centrum Semiovale eGFR = estimated glomerular filtration rate, EPVS = enlarged perivascular spaces.

There were mostly higher rates of severe EPVS (≥11 for the basal ganglia and ≥21 for the centrum semiovale) in those with glomerular hyperfiltration and reduced eGFR, compared with the normofiltration group. The respective rates of severe BG-EPVS and CSO-EPVS were 35% and 26% in the normofiltration group, 45% and 33% in the hyperfiltration group, 45% and 23% in the eGFR 30–60 group, and 49% and 29% in the eGFR <30 group. For full details, see Table 1 and Figure 2.

In ordinal logistic regression models, the associations of hyperfiltration with EPVS in both the basal ganglia and centrum semiovale were significant after adjusting for age, sex, East Asian study center, hypertension, diabetes, hyperlipidemia, ischemic heart disease, and smoking (Table 2). The adjusted estimates suggest that EPVS severity is similar for the normofiltration, eGFR 30–60 and eGFR <30 groups, while it is increased for the hyperfiltration group. For BG-EPVS, compared with normal glomerular filtration, the adjusted odds ratios (95% CIs) for the hyperfiltration, eGFR 30–60 and eGFR <30 groups were 1.38 (1.11–1.70), 1.02 (0.89–1.17) and 1.10 (0.88–1.38), respectively, p for trend <0.001. For CSO-EPVS, the respective adjusted odds ratios (aOR) were 1.34 (1.08–1.64), 0.81 (0.61–1.07), and 0.91 (0.68–1.22), p for trend = 0.011. Individual *p* values for pairwise comparisons between each group and normofiltration are presented in Table 2. In our collinearity evaluation, VIFs for all variables were 1.5 or less, indicating that collinearity does not significantly affect our results.

**Table 2:** Ordinal Logistic Regression Analyses Investigating EPVS Severity According to Glomerular Function

	Basal ganglia EPVS		Centrum semiovale EPVS	
Predictor	Adjusted OR (95% CI)	*p* Value	Adjusted OR (95% CI)	*p* Value
Glomerular filtration category		<0.001		0.011
Normofiltration	Ref.		Ref.	
Hyperfiltration	1.38 (1.11–1.70)	0.003	1.34 (1.08–1.64)	0.006
eGFR 30–60	1.02 (0.89–1.17)	0.800	0.81 (0.61–1.07)	0.143
eGFR <30	1.10 (0.88–1.38)	0.396	0.91 (0.68–1.22)	0.521
Age group		<0.001		0.005
18–50	Ref.		Ref.	
51–60	2.44 (1.60–3.73)		1.45 (1.11–1.90)	
61–70	4.39 (2.11–9.15)		1.61 (1.01–2.56)	
71–80	8.89 (4.34–18.19)		1.90 (1.29–2.79)	
>80	13.83 (6.66–28.71)		1.78 (1.23–2.58)	
Sex	0.86 (0.77–0.96)	0.008	0.84 (0.71–0.98)	0.025
East Asian study center	2.03 (0.87–4.73)	0.101	2.39 (0.73–7.80)	0.150
Hypertension	1.60 (1.36–1.88)	<0.001	1.23 (1.01–1.50)	0.036
Diabetes	0.91 (0.81–1.02)	0.112	1.02 (0.86–1.20)	0.839
Hyperlipidemia	1.16 (0.89–1.52)	0.272	1.14 (0.83–1.59)	0.415
Ischemic heart disease	0.90 (0.72–1.13)	0.361	0.69 (0.52–0.92)	0.013
Current smoker	0.99 (0.85–1.16)	0.922	1.21 (1.06–1.38)	0.005

Abbreviations: eGFR = estimated glomerular filtration rate; EPVS = enlarged perivascular spaces; OR = odds ratio.

### Sensitivity Analyses

The sensitivity analyses were supportive of the primary outcome results. In the multivariable multinomial logistic regression analysis with BG-EPVS as the dependent variable, compared with EPVS 0–10 (the base outcome), the relative risk ratios (RRRs) (95% CIs) of EPVS 11–20 and EPVS ≥21 for the hyperfiltration group were 1.40 (1.03–1.90) and 1.36 (0.95–1.96), respectively. The respective RRRs for the eGFR 30–60 group were 0.93 (0.79–1.10) and 0.95 (0.77–1.18), and for the eGFR <30 group, they were 1.04 (0.82–1.31) and 0.97 (0.66–1.43). In the multivariable multinomial logistic regression analysis for CSO-EPVS, compared with EPVS 0–10, the RRRs (95% CIs) of EPVS 11–20 and EPVS ≥21 for the hyperfiltration group were 1.09 (0.85–1.40) and 1.45 (1.10–1.92), respectively. The respective RRRs for the eGFR 30–60 group were 0.81 (0.61–1.08) and 0.74 (0.52–1.03), and for the eGFR <30 group, they were 0.81 (0.59–1.11) and 0.86 (0.60–1.22). Full models are presented in eTable 2.

In the multivariable logistic regression analysis with severe BG-EPVS (≥11) as the dependent variable, compared with BG-EPVS ≤10 the respective aORs (95% CIs) for hyperfiltration, eGFR 30–60 and eGFR <30 groups were 1.44 (1.12–1.85), 1.01 (0.87–1.16) and 1.11 (0.89–1.39). For the analysis with severe CSO-EPVS (≥21) as the dependent variable, the respective estimates compared with CSO-EPVS ≤20 were 1.39 (1.02–1.90), 0.81 (0.65–1.01), and 0.95 (0.73–1.23).

In the sensitivity analyses where we excluded patients with eGFR >120 mL/minute/1.73m2, there were no substantial changes in the estimates. For BG-EPVS, compared with normofiltration, the respective aORs (95% CIs) for hyperfiltration, eGFR 30–60 and eGFR <30 were 1.40 (1.14–1.73), 1.02 (0.89–1.17), and 1.10 (0.88–1.39). For CSO-EPVS, the respective estimates compared with normofiltration were 1.36 (1.12–1.66), 0.81 (0.61–1.07), and 0.91 (0.68–1.23).

### Subgroup Analyses

**Figure 3:**
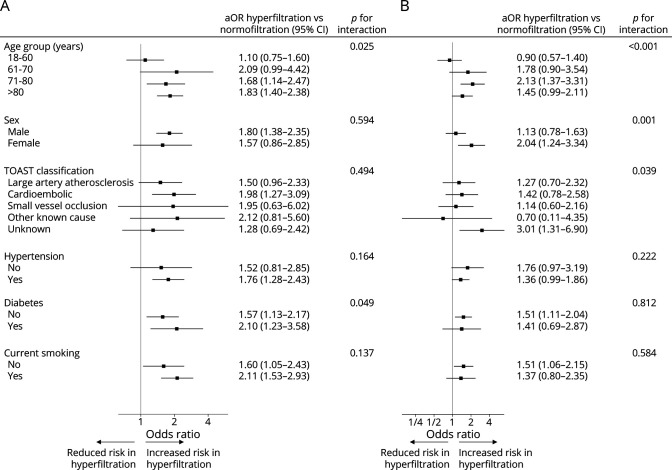
Subgroup Analyses of EPVS Severity in (A) the Basal Ganglia and (B) the Centrum Semiovale According to Glomerular Hyperfiltration aOR = adjusted odds ratio; EPVS = enlarged perivascular space; TOAST = Trial of Org 10172 in Acute Stroke Treatment.

In subgroup analyses, there was a tendency for the association of EPVS with glomerular hyperfiltration to vary according to age group, with no clear association for age 18–60 and significant associations at older age groups. For CSO-EPVS, there was a significant interaction between hyperfiltration and sex, with more severe EPVS in female patients (aOR 2.04, 95% CI 1.24 to 3.34) than male patients (aOR 1.13, 95% CI 0.78 to 1.63). Otherwise, there was no substantial heterogeneity of the observed associations according to index stroke etiology, hypertension, diabetes, or smoking status. Interactions according to hyperfiltration are shown in Figure 3, and the complete subgroup analysis is presented in eTable 3. The subgroup analysis according to study site suggests substantial heterogeneity (eFigure 1).

### Combined cSVD Burden According to Glomerular Filtration

Compared with normal glomerular filtration, the hyperfiltration and both reduced eGFR groups had a worse burden of cSVD, as shown in Table 3 and eFigure 2. In a multivariable ordinal logistic regression model, compared with normal glomerular filtration, eGFR <30 (aOR 1.27, 95% CI 1.03 to 1.57) was independently associated with increased cSVD burden, but eGFR 30–60 (aOR 1.06, 95% CI 0.95 to 1.20) and hyperfiltration (aOR 1.15, 95% CI 0.98 to 1.34) were not (p for trend = 0.002). Individual *p* values for pairwise comparisons between each group and normofiltration are presented in Table 4.

**Table 3:** Cerebral Microbleeds, White Matter Hyperintensities, Lacune Presence, and Combined cSVD Burden According to Glomerular Filtration

	All patients^a^ (n = 5,784)	Hyperfiltration (n = 267)	Normofiltration (n = 4,004)	eGFR 30–60 (n = 1,291)	eGFR <30 (n = 222)
CMB present, (%)	1863 (32.2)	70 (26.2)	1,248 (31.2)	452 (35.0)	93 (41.9)
CMB category, (%)					
0	3,921 (67.8)	197 (73.8)	2,756 (68.8)	839 (65.0)	129 (58.1)
1	800 (13.8)	24 (9.0)	578 (14.4)	165 (12.8)	33 (14.9)
2–4	636 (11.0)	29 (10.9)	399 (10.0)	176 (13.6)	32 (14.4)
>5	427 (7.4)	17 (6.4)	271 (6.8)	111 (8.6)	28 (12.6)
CMB distribution, (%)					
None	3,921 (67.8)	197 (73.8)	2,756 (68.8)	839 (65.0)	129 (58.1)
Strictly lobar	559 (9.7)	16 (6.0)	366 (9.1)	143 (11.1)	34 (15.3)
Strictly nonlobar	687 (11.9)	28 (10.5)	486 (12.1)	150 (11.6)	23 (10.4)
Mixed	617 (10.7)	26 (9.7)	396 (9.9)	159 (12.3)	36 (16.2)
Severe WMH present	2074 (35.9)	109 (40.8)	1,325 (33.1)	541 (41.9)	99 (44.6)
Lacunes present	1709 (29.5)	87 (32.6)	1,120 (28.0)	419 (32.5)	83 (37.4)
cSVD burden score, (%)					
0	1809 (31.3)	79 (29.6)	1,348 (33.7)	337 (26.1)	45 (20.3)
1	1,586 (27.4)	64 (24.0)	1,141 (28.5)	334 (25.9)	47 (21.2)
2	1,183 (20.5)	55 (20.6)	749 (18.7)	311 (24.1)	68 (30.6)
3	825 (14.3)	46 (17.2)	534 (13.3)	204 (15.8)	41 (18.5)
4	381 (6.6)	23 (8.6)	232 (5.8)	105 (8.1)	21 (9.5)
cSVD burden score, median (IQR)	1 (0–2)	1 (0–3)	1 (0–2)	1 (0–2)	2 (1–3)

Abbreviations: CMB = cerebral microbleed; cSVD = cerebral small vessel disease; eGFR = estimated glomerular filtration rate; IQR = interquartile range; WMH = white matter hyperintensities.

a One thousand four hundred seventy excluded as they did not have all the required variables to fully assess cSVD burden.

**Table 4:** Ordinal Logistic Regression Analysis of Combined cSVD Burden Score According to Glomerular Filtration Category

Predictor	Adjusted OR (95% CI)	*p* Value
Glomerular filtration category		0.002
Normofiltration	Ref.	
Hyperfiltration	1.15 (0.98–1.34)	0.087
eGFR 30-60	1.06 (0.95–1.20)	0.302
eGFR <30	1.27 (1.03–1.57)	0.025
Age group		<0.001
18–50	Ref.	
51–60	1.68 (1.35–2.09)	
61–70	2.85 (1.60–5.07)	
71–80	5.03 (2.92–8.68)	
>80	7.65 (4.15–14.12)	
Sex	0.90 (0.77–1.04)	0.154
East Asian study center	2.80 (1.32–5.94)	0.007
Hypertension	1.79 (1.51–2.12)	<0.001
Diabetes	0.98 (0.87–1.10)	0.722
Hyperlipidemia	1.02 (0.82–1.27)	0.838
Ischemic heart disease	0.96 (0.73–1.27)	0.770
Current smoker	1.08 (0.94–1.23)	0.262

Abbreviation: cSVD = cerebral small vessel disease; eGFR = estimated glomerular filtration rate; OR = odds ratio.

In the subgroup analysis of cSVD burden, there was evidence that reduced eGFR predicts cSVD more strongly in younger age groups and those without hypertension. Full details are presented in eTable 4.

### Individual cSVD Markers According to Glomerular Filtration

Compared with normal glomerular filtration, there were higher rates of severe WMH, lacune presence in the hyperfiltration, eGFR 30–60, and eGFR <30 groups. There were lower rates of CMB presence in the hyperfiltration group and higher rates of CMB presence in the reduced eGFR groups. Full details are presented in Table 3. In adjusted analyses, there were statistically significant associations of lacune presence with reduced eGFR and CMB absence with hyperfiltration, but otherwise, associations were not statistically significant. Full details are presented in eTable 5.

## Discussion

In this large international multicenter study of pooled individual patient data, our major findings were that: (1) glomerular hyperfiltration was independently associated with EPVS in both the basal ganglia and the centrum semiovale, but impaired kidney function was not and (2) severe impaired kidney function (eGFR <30), but not glomerular hyperfiltration, had a modest independent association with the combined burden of cSVD. Our findings were broadly consistent in subgroup analyses including index stroke etiology. There was also a modest independent association of reduced eGFR with lacune presence, and a statistically significant lower rate of CMB presence in the glomerular hyperfiltration group. Although glomerular hyperfiltration and reduced eGFR had more severe WMH and those with impaired kidney function had numerically more CMB, these associations were not statistically significant in adjusted analyses.

We are only aware of 3 previous studies investigating EPVS presence and severity according to impaired kidney function in ischemic stroke populations. Xiao et al.^16^ conducted a cross-sectional study (n = 413) and found a significant association of eGFR <60 with BG-EPVS (adjusted OR 4.17, 95% CI 2.08–8.37) and CSO-EPVS (aOR 2.37, 95% CI 1.19 to 4.73). However, confidence intervals are wide reflecting persistent uncertainty, and this study included only participants with lacunar stroke, which partially explains the differing results compared with our study. Lau et al.^14^ recruited unselected ischemic stroke patients in a larger study (n = 959) and found no association of eGFR <60 and BG-EPVS (aOR 0.99, 95% CI 0.71 to 1.38); the authors did not report CSO-EPVS in their results. Similarly, Penton et al.^15^ recruited consecutive patients with ischemic stroke (n = 894) and found no association of CKD with either BG-EPVS (aOR 0.75, 95% CI 0.46 to1.22) or CSO-EPVS (aOR 1.34, 95% CI 0.93 to 1.94). To the best of our knowledge, the only other stroke study investigating this association is a study of consecutive ICH patients^12^ (n = 875), and the authors also found no association of CKD with BG-EPVS (aOR 1.19, 95% CI 0.81 to 1.74). Although CKD has been associated with EPVS severity in a community-based study,^42^ this included only patients with autosomal dominant polycystic kidney disease and matched controls, so population-based differences could account for the differences in comparison with our study.

Our reported association of glomerular hyperfiltration with EPVS burden in both the basal ganglia and centrum semiovale is a novel finding, which might be explained by a number of mechanisms. Glomerular hyperfiltration has been shown to be associated with increased arterial stiffness, independent of hypertension, diabetes, and albuminuria.^43,44^ The mechanisms behind this have not been firmly established, but it is believed that this might be related to overstimulation of the renin-angiotensin-aldosterone (RAA) axis which is known to occur in hyperfiltration.^29,45,46^ Arterial stiffness has been associated with EPVS in community-based populations using both a visual rating approach^47^ and volumetric perivascular space quantification.^48^ Overactivity of the RAA pathway has also been demonstrated to increase blood-brain barrier permeability in animal models,^49^ and in turn, increased BBB permeability has been associated with EPVS.^3^

It is not straightforward to explain why EPVS were associated with glomerular hyperfiltration, an early marker of kidney dysfunction, and not overt reduced eGFR. As EPVS are cSVD manifestations which appear relatively early in the disease process, it is possible that this reflects the earlier development of glomerular hyperfiltration in the natural history of microvascular kidney and cerebral damage. In addition, the eGFR 30–60 and <30 groups were significantly older than the normal glomerular function group, with higher rates of common comorbidities, whereas the characteristics of the hyperfiltration and normofiltration groups were relatively similar. Therefore, in adjusted analyses, any associations of impaired kidney function with EPVS will be diluted to a greater extent by confounder adjustment. It is also possible that CKD-related disease processes which are known to involve hyperfiltration in their natural history, such as diabetes, hypertension, and obesity-related microvasculature damage, contribute to the development of EPVS, whereas other CKD causes such as ischemic kidney disease do not.

Associations of impaired kidney function and total MR-visible cSVD burden have been investigated in 1 unselected ischemic stroke population,^14^ 2 lacunar stroke populations,^16,18^ and 2 ICH populations.^12,17^ No association was found in the unselected ischemic stroke study, whereas in the other 4 cSVD-related stroke populations, significant independent associations were found, with adjusted odds ratios (95% CIs) ranging from 1.83 (1.31–2.56) to 5.59 (2.58–12.08). This is to be expected, and it is possible that impaired kidney function plays more of a role in the pathophysiology of cSVD in populations where cSVD is specifically the cause of stroke, which have more severe baseline cSVD. This could be a reason that we found a more modest association of impaired kidney function with cSVD burden, compared with the 4 above studies of cSVD-related stroke populations. In subgroup analysis, eGFR <60 predicted substantially increased risk of cSVD in the younger age group (18–60 years), and eGFR <30 predicted substantially increased risk in those without hypertension. Since the age of those with eGFR <60 in the MICON population is high (mean 77.5 years), this population characteristic could also account in part for our modest observed association with reduced eGFR and cSVD burden. Indeed, a UK-based study of patients with stroke or TIA found an independent association of reduced eGFR with cSVD burden, but only in those younger than 60 years.^50^

Glomerular hyperfiltration was not independently associated with cSVD burden in our study, although the raw proportions suggest that there could be a modest effect (46% with a score ≥2 vs 38% for normofiltration, unadjusted ordinal odds ratio 1.37). As the hyperfiltration group was smaller for the subgroup of patients with data available on all the necessary cSVD markers (n = 267), this analysis did not have as much statistical power as the EPVS analysis to detect a difference according hyperfiltration. In addition, the rate of CMB presence was lower in the hyperfiltration group than the normofiltration, eGFR 30–60 and eGFR <30 groups (26%, 31%, 35%, and 42%, respectively), suggesting that CMB might develop later in the course of glomerular damage. This effect has diluted any potential association of hyperfiltration with cSVD burden in this study. These findings seem to be at odds with our EPVS results. Although different individual cSVD biomarkers have shared risk factors, the pathophysiology remains incompletely understood, and it could be that glomerular hyperfiltration (an early stage of glomerular injury) identifies earlier cerebral microvascular damage causing EPVS, but other cSVD markers are related to other risk factors such as hypertension and CKD. Our results should be considered as hypothesis-generating and further research is needed to clarify these findings.

Our study's findings, if replicated in future studies, could have clinical relevance. They are supportive of the hypothesis that cSVD is part of a multisystem disorder. Glomerular hyperfiltration can lead to albuminuria, and increased blood-brain barrier permeability, as evidenced by EPVS, could be an analogous process in the brain. Identifying hyperfiltration in clinical practice could provide an opportunity for earlier intervention. Patients with early kidney dysfunction, albuminuria or high eGFR could be screened with brain MRI, particularly if they have neurologic or cognitive symptoms. If EPVS or other cSVD markers are found, this could identify a group of patients that would benefit from escalated vascular prevention treatments, particularly blood pressure management.

Our study has several methodological strengths including its large sample size and wide geographical reach, increasing the generalizability of our results. To the best of our knowledge, there is no existing study investigating EPVS severity according to kidney function with a comparable sample size, and therefore, our estimates are more precise than those of previous studies. There are limitations which we acknowledge. First, the observational design means that despite multivariable adjustment we cannot exclude residual confounding from unmeasured between-group differences. Second, our assessment of glomerular function comes from a single blood test at stroke onset, so we cannot reliably exclude acute kidney injury (AKI) which introduces possible measurement error into our results. Elevated eGFR could be caused by artefacts such as frailty or sarcopenia, and since stroke is an acute illness (with high rates of AKI) in some cases, the data recorded will not reflect the patient's baseline eGFR. However, physiologic stresses such as sepsis, volume depletion, or heart failure will cause eGFR to fall, so although our definition of hyperfiltration will cause us to miss cases (i.e., lacks sensitivity), those with high eGFR are likely to truly have it (i.e., our definition should be specific). In addition, our sensitivity analysis excluding those with extremely high eGFR was supportive of our results. Third, we did not have data on albuminuria or body mass index, so we could not adjust for these important confounders. We acknowledge that these are significant limitations, since albuminuria has previously been consistently associated with cSVD and stroke risk, and obesity is an important cause of hyperfiltration. In addition, we did not have access to more sensitive biomarkers of kidney dysfunction such as cystatin C. Fourth, in this large collaborative study, we only had EPVS data from visual rating scores. Quantitative data on EPVS volume might have greater power to detect associations with kidney function. Finally, our associations of EPVS severity with hyperfiltration showed substantial heterogeneity according to study site. These limitations, along with the lack of an association of EPVS with more advanced renal dysfunction in our study, suggest that our results should be interpreted with caution, and further studies are needed, ideally using volumetric assessment of EPVS.

In conclusion, we report a novel independent association of glomerular hyperfiltration with EPVS in both the basal ganglia and centrum semiovale. Specific microvascular pathologies affecting glomerular and cerebral arterioles could be responsible for this effect, and further research is needed to confirm these findings, and potentially develop treatments for systemic small vessel pathology.

## Glossary

AKI: acute kidney injury
aOR: adjusted odds ratio
BG-EPVS: basal ganglia-EPVS
CKD: chronic kidney disease
CMB: cerebral microbleed
CSO-EPVS: centrum semiovale
cSVD: cerebral small vessel disease
eGFR: estimated glomerular filtration rate
EPVS: enlarged perivascular space
MICON: Microbleeds International Collaborative Network
RRR: relative risk ratio
VIF: variance inflation factor
WMH: white matter hyperintensity

## References

[1] Duering M, Biessels GJ, Brodtmann A, et al. Neuroimaging standards for research into small vessel disease-advances since 2013. Lancet Neurol. 2023;22(7):602-618. doi:10.1016/s1474-4422(23)00131-x 37236211

[2] Nedergaard M, Goldman SA. Glymphatic failure as a final common pathway to dementia. Science. 2020;370(6512):50-56. doi:10.1126/science.abb8739 33004510 PMC8186542

[3] Li Y, Li M, Yang L, et al. The relationship between blood-brain barrier permeability and enlarged perivascular spaces: a cross-sectional study. Clin Interventions Aging. 2019;14:871-878. doi:10.2147/cia.s204269 PMC6519012 31190773

[4] Francis F, Ballerini L, Wardlaw JM. Perivascular spaces and their associations with risk factors, clinical disorders and neuroimaging features: a systematic review and meta-analysis. Int J Stroke. 2019;14(4):359-371. doi:10.1177/1747493019830321 30762496

[5] Best JG, Ambler G, Wilson D, et al. Clinical associations and prognostic value of MRI-visible perivascular spaces in patients with ischemic stroke or TIA: a pooled analysis. Neurology. 2024;102(1):e207795. doi:10.1212/wnl.0000000000207795 38165371 PMC10834118

[6] Charidimou A, Jaunmuktane Z, Baron JC, et al. White matter perivascular spaces: an MRI marker in pathology-proven cerebral amyloid angiopathy?. Neurology. 2014;82(1):57-62. doi:10.1212/01.wnl.0000438225.02729.04 24285616 PMC3873625

[7] Charidimou A, Boulouis G, Frosch MP, et al. The Boston criteria version 2.0 for cerebral amyloid angiopathy: a multicentre, retrospective, MRI-Neuropathology diagnostic accuracy study. Lancet Neurol. 2022;21(8):714-725. doi:10.1016/s1474-4422(22)00208-3 35841910 PMC9389452

[8] Lei H, Wu X, Ambler G, et al. Association between perivascular spaces burden and future stroke risk in ischemic stroke and transient ischemic attack: a systematic review and meta-analysis. Eur Neurol. 2024;87(3):130-139. doi:10.1159/000539730 38981445

[9] Kelly DM, Rothwell PM. Does chronic kidney disease predict stroke risk independent of blood pressure?: a systematic review and meta-regression. Stroke. 2019;50(11):3085-3092. doi:10.1161/strokeaha.119.025442. 31594463 PMC6824504

[10] Molad J, Miwa K, Nash PS, et al. Increased risk of recurrent stroke in patients with impaired kidney function: results of a pooled analysis of individual patient data from the MICON international collaboration. J Neurol Neurosurg Psychiatry. 2025;96(9):842-851. doi:10.1136/jnnp-2024-335110 40274401 PMC12418584

[11] Vanent KN, Leasure AC, Acosta JN, et al. Association of chronic kidney disease with risk of intracerebral hemorrhage. JAMA Neurol. 2022;79(9):911-918. doi:10.1001/jamaneurol.2022.2299 35969388 PMC9379821

[12] Nash PS, Fandler-Hofler S, Ambler G, et al. Associations of cerebral small vessel disease and chronic kidney disease in patients with acute intracerebral hemorrhage: a cross-sectional study. Neurology. 2024;103(2):e209540. doi:10.1212/wnl.0000000000209540 38889380 PMC11254447

[13] Xiao CY, Ma YH, Ou YN, et al. Association between kidney function and the burden of cerebral small vessel disease: an updated meta-analysis and systematic review. Cerebrovasc Dis. 2023;52(4):376-386. doi:10.1159/000527069 36599326

[14] Lau KK, Tsang ACO, Teo KC, et al. Age-specific associations of renal impairment and cerebral small vessel disease burden in Chinese with ischaemic stroke. J Stroke Cerebrovasc Dis. 2019;28(5):1274-1280. doi:10.1016/j.jstrokecerebrovasdis.2019.01.018 30853188

[15] Penton AA, Lau H, Babikian VL, et al. Chronic kidney disease as risk factor for enlarged perivascular spaces in patients with stroke and relation to racial group. Stroke. 2020;51(11):3348-3351. doi:10.1161/strokeaha.119.028688 33019895 PMC7606816

[16] Xiao L, Lan W, Sun W, et al. Chronic kidney disease in patients with lacunar stroke: association with enlarged perivascular spaces and total magnetic resonance imaging burden of cerebral small vessel disease. Stroke. 2015;46(8):2081-2086. doi:10.1161/strokeaha.114.008155 26138123

[17] Xu XH, Ye XH, Cai JS, et al. Association of renal dysfunction with remote diffusion-weighted imaging lesions and total burden of cerebral small vessel disease in patients with primary intracerebral hemorrhage. Front Aging Neurosci. 2018;10:171. doi:10.3389/fnagi.2018.00171 29930507 PMC6001158

[18] Yang S, Cai J, Lu R, Wu J, Zhang M, Zhou X. Association between serum cystatin C level and total magnetic resonance imaging burden of cerebral small vessel disease in patients with acute lacunar stroke. J Stroke Cerebrovasc Dis. 2017;26(1):186-191. doi:10.1016/j.jstrokecerebrovasdis.2016.09.007 27727072

[19] Lee BC, Tsai HH, Chen ZW, et al. Aldosteronism is associated with more severe cerebral small vessel disease in hypertensive intracerebral hemorrhage. Hypertens Res. 2024;47:608-617. doi:10.1038/s41440-023-01458-w 37993592

[20] Doubal FN, MacLullich AM, Ferguson KJ, Dennis MS, Wardlaw JM. Enlarged perivascular spaces on MRI are a feature of cerebral small vessel disease. Stroke. 2010;41(3):450-454. doi:10.1161/strokeaha.109.564914 20056930

[21] Nash PS, Fandler-Hofler S, Ambler G, et al. Associations of chronic kidney disease with recurrent stroke in patients with intracerebral haemorrhage. Eur Stroke J. 2026;11(1):aakaf007. doi:10.1093/esj/aakaf007 41614490 PMC12866628

[22] Ito S, Nagasawa T, Abe M, Mori T. Strain vessel hypothesis: a viewpoint for linkage of albuminuria and cerebro-cardiovascular risk. Hypertens Res. 2009;32(2):115-121. doi:10.1038/hr.2008.27 19262469

[23] Kadry H, Noorani B, Cucullo L. A blood-brain barrier overview on structure, function, impairment, and biomarkers of integrity. Fluids Barriers CNS. 2020;17(1):69. doi:10.1186/s12987-020-00230-3 33208141 PMC7672931

[24] Haraldsson B, Nystrom J. The glomerular endothelium: new insights on function and structure. Curr Opin Nephrol Hypertens. 2012;21(3):258-263. doi:10.1097/mnh.0b013e3283522e7a. 22388551

[25] Cortinovis M, Perico N, Ruggenenti P, Remuzzi A, Remuzzi G. Glomerular hyperfiltration. Nat Rev Nephrol. 2022;18(7):435-451. doi:10.1038/s41581-022-00559-y 35365815

[26] Dupuis ME, Nadeau-Fredette AC, Madore F, Agharazii M, Goupil R. Association of glomerular hyperfiltration and cardiovascular risk in middle-aged healthy individuals. JAMA Netw Open. 2020;3(4):e202377. doi:10.1001/jamanetworkopen.2020.2377 32275320 PMC7148438

[27] Eriksen BO, Lochen ML, Arntzen KA, et al. Subclinical cardiovascular disease is associated with a high glomerular filtration rate in the nondiabetic general population. Kidney Int. 2014;86(1):146-153. doi:10.1038/ki.2013.470 24304885

[28] Inrig JK, Gillespie BS, Patel UD, et al. Risk for cardiovascular outcomes among subjects with atherosclerotic cardiovascular disease and greater-than-normal estimated glomerular filtration rate. Clin J Am Soc Nephrol. 2007;2(6):1215-1222. doi:10.2215/cjn.00930207 17942781

[29] Helal I, Fick-Brosnahan GM, Reed-Gitomer B, Schrier RW. Glomerular hyperfiltration: definitions, mechanisms and clinical implications. Nat Rev Nephrol. 2012;8(5):293-300. doi:10.1038/nrneph.2012.19 22349487

[30] Wetzels JF, Kiemeney LA, Swinkels DW, Willems HL, Heijer MD. Age- and gender-specific reference values of estimated GFR in Caucasians: the nijmegen biomedical study. Kidney Int. 2007;72(5):632-637. doi:10.1038/sj.ki.5002374 17568781

[31] KDIGO Kidney Working Group. KDIGO 2012 Clinical Practice Guideline for the Evaluation and Management of Chronic Kidney Disease. Accessed July 22, 2025. kdigo.org/wp-content/uploads/2017/02/KDIGO_2012_CKD_GL.pdf2013

[32] Fu EL, Coresh J, Grams ME, et al. Removing race from the CKD-EPI equation and its impact on prognosis in a predominantly White European population. Nephrol Dial Transplant. 2023;38(1):119-128. doi:10.1093/ndt/gfac197 35689668 PMC9869854

[33] Gansevoort RT, Anders HJ, Cozzolino M, et al. What should European nephrology do with the new CKD-EPI equation? Nephrol Dial Transplant. 2023;38:1-6. doi:10.1093/ndt/gfac254 36069913 PMC9869851

[34] Delanaye P, Schaeffner E, Cozzolino M, et al. The new, race-free, chronic kidney disease epidemiology consortium (CKD-EPI) equation to estimate glomerular filtration rate: is it applicable in Europe? A position statement by the european Federation of clinical hemistry and laboratory medicine (EFLM). Clin Chem Lab Med. 2023;61(1):44-47. doi:10.1515/cclm-2022-0928 36279207

[35] Potter GM, Chappell FM, Morris Z, Wardlaw JM. Cerebral perivascular spaces visible on magnetic resonance imaging: development of a qualitative rating scale and its observer reliability. Cerebrovasc Dis. 2015;39(3-4):224-231. doi:10.1159/000375153 25823458 PMC4386144

[36] Potter GMMZ, Wardlaw Joanna M. Enlarged Perivascular Spaces (EPVS): A Visual Rating Scale and User Guide. Accessed July 23, 2025. ed.ac.uk/files/imports/fileManager/epvs-rating-scale-user-guide.pdf

[37] Fazekas F, Chawluk JB, Alavi A, Hurtig HI, Zimmerman RA. MR signal abnormalities at 1.5 T in Alzheimer's dementia and normal aging. Am J Roentgenology. 1987;149(2):351-356. doi:10.2214/ajr.149.2.351 3496763

[38] Wahlund LO, Barkhof F, Fazekas F, et al. A new rating scale for age-related white matter changes applicable to MRI and CT. Stroke. 2001;32(6):1318-1322. doi:10.1161/01.str.32.6.1318 11387493

[39] Gregoire SM, Chaudhary UJ, Brown MM, et al. The microbleed anatomical rating scale (MARS): reliability of a tool to map brain microbleeds. Neurology. 2009;73(21):1759-1766. doi:10.1212/wnl.0b013e3181c34a7d 19933977

[40] Staals J, Makin SD, Doubal FN, Dennis MS, Wardlaw JM. Stroke subtype, vascular risk factors, and total MRI brain small-vessel disease burden. Neurology. 2014;83(14):1228-1234. doi:10.1212/wnl.0000000000000837 25165388 PMC4180484

[41] Adams HP Jr., Bendixen BH, Kappelle LJ, et al. Classification of subtype of acute ischemic stroke. Definitions for use in a multicenter clinical trial. TOAST. Trial of org 10172 in acute stroke treatment. Stroke. 1993;24(1):35-41. doi:10.1161/01.str.24.1.35 7678184

[42] Lee WJ, Jung KH, Ryu H, et al. Association of autosomal dominant polycystic kidney disease with cerebral small vessel disease. J Cereb Blood Flow Metab. 2021;41(12):3365-3377. doi:10.1177/0271678x211037869 34415212 PMC8669289

[43] Lin L, Peng K, Du R, et al. High glomerular filtration rate is associated with arterial stiffness in Chinese population. J Hypertens. 2017;35(2):385-391. doi:10.1097/hjh.0000000000001158 28005707

[44] Yang E, Park SH, Lee S, et al. Pulse pressure and the risk of renal hyperfiltration in young adults: results from Korea national health and nutrition examination survey (2010-2019). Front Med (Lausanne). 2022;9:911267. doi:10.3389/fmed.2022.911267 36177333 PMC9513024

[45] Sochett EB, Cherney DZ, Curtis JR, Dekker MG, Scholey JW, Miller JA. Impact of renin angiotensin system modulation on the hyperfiltration state in type 1 diabetes. J Am Soc Nephrol. 2006;17(6):1703-1709. doi:10.1681/asn.2005080872 16672313

[46] Fesler P, Mourad G, du Cailar G, Ribstein J, Mimran A. Arterial stiffness: an independent determinant of adaptive glomerular hyperfiltration after kidney donation. Am J Physiology-Renal Physiol. 2015;308(6):F567-F571. doi:10.1152/ajprenal.00524.2014 25568135

[47] Ariko TA, Aimagambetova B, Gardener H, et al. Estimated pulse-wave velocity and magnetic resonance imaging markers of cerebral small-vessel disease in the NOMAS. J Am Heart Assoc. 2024;13(15):e035691. doi:10.1161/jaha.124.035691 39023069 PMC11964018

[48] Bjornfot C, Eklund A, Larsson J, et al. Cerebral arterial stiffness is linked to white matter hyperintensities and perivascular spaces in older adults - a 4D flow MRI study. J Cereb Blood Flow Metab. 2024;44(8):1343-1351. doi:10.1177/0271678x241230741 38315044 PMC11342729

[49] Makuch-Martins M, Vieira-Morais CG, Perego SM, Ruggeri A, Ceroni A, Michelini LC. Angiotensin II, blood-brain barrier permeability, and microglia interplay during the transition from pre-to hypertensive phase in spontaneously hypertensive rats. Front Physiol. 2024;15:1452959. doi:10.3389/fphys.2024.1452959 39328833 PMC11425344

[50] Liu B, Lau KK, Li L, et al. Age-specific associations of renal impairment with magnetic resonance imaging markers of cerebral small vessel disease in transient ischemic attack and stroke. Stroke. 2018;49(4):899-904. doi:10.1161/strokeaha.117.019650 29523652 PMC5895118

